# Classification and Characteristics of Pain Associated with Parkinson's Disease

**DOI:** 10.1155/2016/6067132

**Published:** 2016-10-05

**Authors:** Marcelo Rezende Young Blood, Marcelo Machado Ferro, Renato Puppi Munhoz, Hélio Afonso Ghizoni Teive, Carlos Henrique Ferreira Camargo

**Affiliations:** ^1^Neurology Service, Hospital Universitário, State University of Ponta Grossa, Ponta Grossa, PR, Brazil; ^2^Neuropsychopharmacology Laboratory, State University of Ponta Grossa, Ponta Grossa, PR, Brazil; ^3^Movement Disorders Centre, Toronto Western Hospital, University of Toronto, Toronto, ON, Canada; ^4^Movement Disorders Unit, Neurology Service, Internal Medicine Department, Hospital de Clínicas, Federal University of Paraná, Curitiba, PR, Brazil

## Abstract

Neuropsychiatric symptoms and pain are among the most common nonmotor symptoms of Parkinson's disease (PD). The correlation between pain and PD has been recognized since its classic descriptions. Pain occurs in about 60% of PD patients, two to three times more frequent in this population than in age matched healthy individuals. It is an early and potentially disabling symptom that can precede motor symptoms by several years. The lower back and lower extremities are the most commonly affected areas. The most used classification for pain in PD defines musculoskeletal, dystonic, central, or neuropathic/radicular forms. Its different clinical characteristics, variable relationship with motor symptoms, and inconsistent response to dopaminergic drugs suggest that the mechanism underlying pain in PD is complex and multifaceted, involving the peripheral nervous system, generation and amplification of pain by motor symptoms, and neurodegeneration of areas related to pain modulation. Although pain in DP is common and a significant source of disability, its clinical characteristics, pathophysiology, classification, and management remain to be defined.

## 1. Introduction

Although the most commonly accepted criteria for the diagnosis of Parkinson's disease (PD) depend on motor symptoms, other signs and complaints related to the disease, such as a loss of sense of smell, constipation, depression, altered sleep patterns, and unexplained pain, may be detected years before the onset of any of the cardinal motor signs [1]. One landmark study showed that virtually all PD patients report at least one nonmotor symptom (NMS) when these manifestations are actively pursued using specific questionnaires, reaching an average of eight different symptoms per patient. In this same study, the most frequent NMS were neuropsychiatric, gastrointestinal, and pain related symptoms [2]. In the early stages of the disease, the most common NMS are hyposmia, pain, and sleep disturbances [3]. Additionally, pain, depression, and anxiety are associated with worse quality of life [3–5]. Although the correlation between pain and PD has been recognized since its first descriptions, including James Parkinson citation of “rheumatic pain extending from the arms to the fingers” [6], the issue of pain in PD has only recently received the deserved attention in both clinical and research settings.

Pain is present in around 60% of PD patients and occurs two to three times more frequently in this population than in age matched individuals without PD [7–9]. It is an early symptom and can precede motor symptoms by several years [1, 10, 11]. In individuals who have had the disease for less than six years, it can be the most disturbing NMS, particularly when it has musculoskeletal or visceral origin [12]. Although common in any disease stage [12], around 40% of patients may not report this complaint in routine visits to a physician [13].

Pain does not appear to be influenced by gender, age, or geographic/cultural variables [14]. Although some authors have reported a higher frequency in women [2, 7], this is not supported by most studies [8, 15–17]. Similarly, while some studies have reported a lower mean age for patients with pain [9, 15] and others have even suggested that onset of PD before the age of 65 is a risk factor for pain [9], most authors [8, 17–19] failed to find significant differences. An increase in the prevalence of pain should also be expected throughout the clinical and neurodegenerative progression of the disease; however, most studies showed no correlation between the presence or intensity of pain and clinical staging or disease duration [7, 19–21].

Pain usually occurs on the side on which motor symptoms first appear or are more severe [22]. While the main risk factors for developing spontaneous pain in PD are the presence of motor fluctuations and severity of motor symptoms [9, 16], this may not always be the case [23], as some patients with pain and motor complications present with pain on the side unaffected by the disease [16]. In a study of the influence of levodopa treatment and deep brain stimulation (DBS) on the “off” period in PD, both interventions improved somatic pain thresholds independently of any improvement in motor symptoms [24]. In terms of pain location, although it can be quite variable, the lower back and legs are the most commonly affected regions [14]. Shoulder pain, commonly reported by PD patients [2, 16], can be the first symptom of PD in 2–8% of patients and might even precede the onset of motor symptoms [25]. It is thought to stem from two different mechanisms: one, directly related to the neurological symptom, is pseudorheumatic and dopamine sensitive; the other is thought to be associated with degenerative lesions that may worsen with the progression of PD [18].

As depression and pain are prevalent NMS in PD and chronic pain is a known risk factor for depression [39], the association between the two symptoms is to be expected [40]. Indeed, individuals with pain and PD have higher scores on depression rating scales [41]. Although some studies failed to find any association [16, 17], current evidence suggests that the presence of depression is an important variable that should be taken into account in studies of pain in PD, particularly because of the frequency with which it is found and the fact that it can modify the perception of pain. Furthermore, it is potentially treatable [40].

Systemic diseases such as diabetes mellitus, osteoporosis, and rheumatic diseases are also associated with a greater prevalence of pain in PD [42]. Finally, there is also evidence of an association between genetic factors and musculoskeletal pain, a subtype of pain in PD. For example, mutations in the* SCN9A* (sodium channel Nav1.7) and* FAAH* (fatty acid amide hydrolase, a key cannabinoid metabolizing enzyme) genes have been associated with higher susceptibility to this symptom in PD [43, 44].

## 2. Assessment and Classification of Pain in PD

The prevalence of pain in PD can vary from 34% [45] to 83% [7] depending on methodological assessments. Possible explanations for this large range include the tools and criteria used for the diagnosis and profile of the population studied. For instance, while some studies only considered this symptom to be present when it lasted more than three months [8, 9, 17, 46], others did not specify any time criterion [18, 19, 47]. Among the diagnostic tools for the assessment of pain in PD, the most widely used is the Brief Pain Inventory [5, 7, 9, 19–21]; however, because it only analyzes pain in the previous 24 hours, it can underestimate its prevalence. As pain is an unpleasant sensory and emotional experience [48], the use of a multidimensional scale to evaluate it is recommended [49]. The widely used McGill Pain Questionnaire [9, 18, 47, 50] is an example of this type of scale, evaluating sensory-discriminatory and affective-motivational domains [51].

Nonetheless, there is a lack of consensus regarding the assessment and classification of pain in PD patients. The first classification, published by Ford [52], is the most commonly used, but other proposals have been put forward as a result of the growing interest in the subject [9, 25, 53, 54].

### 2.1. The Ford Classification

The Ford classification [52] is based on studies by Goetz and Quinn [15, 55] and uses an approach that involves the etiology of pain and its association with motor symptoms (Table 1). It classifies PD related pain in five groups: musculoskeletal, dystonic, neuropathic/radicular, central or primary, and akathisia.

Musculoskeletal pain is the most common and easily identified type of pain [11]. It is associated with muscle rigidity and bradykinesia as well as a lack of mobility, postural abnormalities, and gait imbalance [56]. Lumbar pain is usually the most frequent complaint, but neck and leg pain are also common. Joint pain is most common in the shoulders, hips, knees, and ankles [56]. In cases of stiffness-related pain, adjustment of dopaminergic therapy together with physiotherapy and physical exercise is recommended to relieve symptoms. Nonsteroidal anti-inflammatory drugs and analgesics can help in orthopedic and rheumatologic conditions.

Dystonia is a movement disorder characterized by sustained or intermittent muscle contractions that result in abnormal, frequently repetitive movements and/or postures [57]. These contractions can cause the most intense pains PD patients experience and can be alleviated by intramuscular botulinum toxin injections as well as adjustment of dopaminergic therapy [58]. It is important to try to correlate the timing of dopaminergic therapy with the occurrence of pain as it can be observed as an early morning or as wearing off (WO) phenomenon, indicating dopaminergic deficiency [56]. Treatment with DBS of the subthalamic nucleus or internal globus pallidus may influence this symptom positively [59, 60].

Very localized pain with neuropathic characteristics (burning, electric-shock like, and paresthesia) limited to a nerve or nerve root territory was classified by Ford as neuropathic/radicular and can also be called peripheral neuropathic pain. It affects from 5% to 14% of PD patients with pain [9, 15, 19]. To our knowledge, there are no studies that have evaluated only peripheral neuropathic pain in PD; however, in most PD patients this type of pain is believed to be associated with focal compression associated with degenerative joint disease [56, 61] and assessments with electroneuromyography and neuroimaging may be necessary. The first-line drug treatments for neuropathic pain are amitriptyline, duloxetine, and pregabalin [62].

Central or primary pain presents with neuropathic characteristics and appears to be associated with impaired central modulation of pain caused by dopaminergic deficiency in the basal ganglia. It affects from 4% to 10% of PD patients with pain [7, 8]. It is typically not restricted to a nerve territory and has been described to affect atypical body areas such as face, head, pharynx, epigastrium, abdomen, pelvis, rectum, and genitalia [63]. This type of pain can be associated with autonomic manifestations, with visceral sensations, and can improve with administration of levodopa [52]. As it cannot be classified in any other group, it is considered a diagnosis of exclusion.

Akathisia is defined as a feeling of inner restlessness and an inability to remain still, presenting as a constant need to move or change position. Although it is sometimes described as a painful sensation, this is not normally the case and it should not be considered a sensory disturbance [7]. Javoy-Agid and Agid [64] suggest that akathisia is a result of a dopamine dysfunction in the dopaminergic mesocorticolimbic pathway, which originates in the ventral tegmental area and is impaired in PD. Restless legs syndrome is also caused by a similar dopamine dysfunction in this region, and both conditions respond to dopaminergic therapy [65].

### 2.2. Other Assessment Tools

The DoPaMiP study (*Douleur et maladie de Parkinson en Midi-Pyrénées*) suggests that an initial distinction can be made between pain related to PD (PRPD) and pain unrelated to PD (PUPD). This distinction was made by an expert group who based their conclusions on the relationship between pain and PD as reported by the patient, the clinical characteristics of the pain (such as location, duration, and frequency), aggravating factors and the association between pain and the disease symptoms, motor complications, and anti-Parkinsonian medication. PRPD can be further divided into (1) pain directly related to PD, when it cannot be attributed to any other health problem, and (2) pain indirectly related to PD, when it is caused by another disease (e.g., osteoarthritis) but is aggravated by PD because of rigidity and abnormal posture or movements. Hence, to be considered PRPD, the pain must be caused or aggravated by PD, while for it to be considered PUPD it should be related to other causes and not aggravated by PD [9].

The Marburg/São Paulo/Créteil Questionnaire uses a three-step approach. These consist of (1) establishing a temporal relation between pain and the symptoms of PD; (2) determining whether the pain is related to motor fluctuations; and (3) determining whether the pain is related to the anti-Parkinsonian treatment. If the answer in any of these three cases is affirmative, the pain is classified as PRPD; if not, it is classified as PUPD. In the former case, it can be further classified as musculoskeletal, neuropathic, or psychomotor/restlessness-related.

In some cases, the relation between pain and PD is difficult to determine. Despite obvious injury on imaging studies the cause and effect relationship can not be easily observed (e.g., disc herniation and low back pain). In DoPaMiP study, for example, patients with osteoarthritis were classified as either PRPD or PUPD. The authors agree that selection bias may have occurred in this approach [9]. There are two disadvantages to this approach: it does not define any subtype of pain and does not provide the individual response to dopaminergic therapy [66]. The perception of pain and other sensory modalities is clearly altered in individuals with PD [67], so it is not necessary to separate the pain related or unrelated to the disease.

“King's Parkinson's disease pain scale,” which was proposed by a multicenter group that included King's College Hospital in London, is officially advocated by the “International Parkinson's and Movement Disorder Society Non-Motor PD Study Group” for evaluating pain in PD. It is a questionnaire with fourteen questions covering seven domains: (1) musculoskeletal pain; (2) chronic pain; (3) fluctuation-related pain; (4) nocturnal pain; (5) orofacial pain; (6) discoloration and edema/swelling; and (7) radicular pain. For each question, scores from 0 to 3 and 0 to 4 are assigned for the intensity and frequency of the symptom, respectively. The scores for severity and frequency are multiplied and the result is added to the result of the other items to obtain a score for each domain and, in the same way, the total score for the seven domains (0–168). The validation study found a strong correlation between this scale's scores and the Parkinson's disease-severity and quality of life scales scores. According to the authors, the score in each domain should be used to determine the type of pain the patient is presenting with, while the total score provides insight of the impact pain has on the individual's life [53]. This is a new approach to pain in PD; although it may be too long to complete in routine clinical care, it will allow further in-depth testing in clinical trials for treatments of this specific aspect of PD.

## 3. The Pathophysiology of Pain in Parkinson's Disease

**Figure 1:**
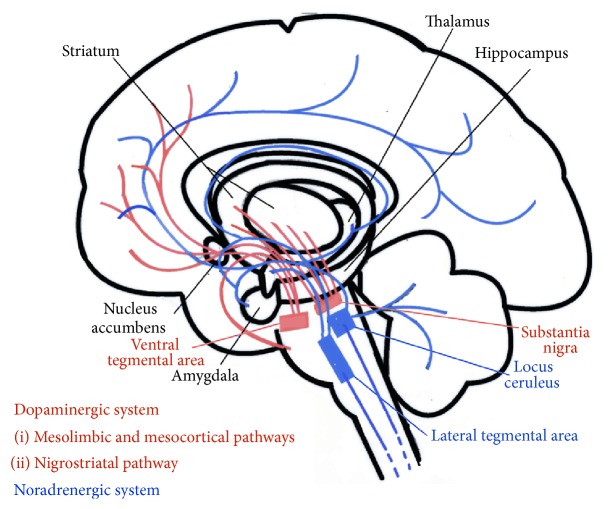
Dopaminergic and noradrenergic ascending pathways. This figure was drawn by the authors from information taken from texts and figures in literature [26–38].

Motor symptoms in PD are mainly the result of degeneration of dopaminergic neurons in the substantia nigra pars compacta, affecting the nigrostriatal pathway and reducing stimulation of the motor cortex by the basal nuclei and thalamus (Figure 1). The pathological hallmark of the disease is the presence of Lewy bodies, which are spherical, eosinophilic, cytoplasmic inclusions in the degenerated neurons, containing mainly *α*-synuclein and ubiquitin. However, the changes are not restricted to this brain area and can be found in other nuclei of the brainstem and cortex and even peripheral neurons, such as those in the myenteric plexus [68]. Analysis of the distribution of these pathological markers in PD by Braak et al. [68] allowed the understanding of both motor and NMS of the disease through a logical six-stage ascending progression in a caudorostral direction. According to this hypothesis, the degenerative process starts in the caudal regions of the brainstem, such as the dorsal motor nuclei of the glossopharyngeal and vagus nerves and the anterior olfactory nucleus, passing through the locus coeruleus and Meynert's basal nucleus, the dorsal raphe nuclei, amygdalae, and hypothalamus and progressing through practically the whole cerebral cortex [68–70]. Motor symptoms are apparent in stage 3, after the mesencephalon has been affected. Prior to this stage, degeneration of the dorsal motor nucleus of the vagus (stage 1) can help to explain certain autonomic complaints such as obstipation and hypotension, while serotoninergic and noradrenergic dysfunction of the raphe nuclei and locus coeruleus, respectively (stage 2), can explain part of the pain related symptoms that occur in early PD [26, 27]. The exact mechanism by which cell degeneration occurs has yet to be elucidated but is thought to involve a cascade of events including interaction between genetic and environmental factors, impaired protein processing, oxidative stress, mitochondrial dysfunction, excitotoxicity, abnormal immune regulation, and inflammatory responses [28].

The basal nuclei play a central role in modulation of various pathways and function as components of a series of parallel circuits related to the motor, limbic, and associative systems (Figure 1). Striatal neurons can respond to different sensory stimuli applied to various body parts [29]. Of the four main dopaminergic pathways, two produce most of the dopamine in the brain: the nigrostriatal pathway, which is directly involved in PD, and the mesolimbic pathway, which is related to the reward system and central modulation of pain. The latter connects the ventral tegmental area of the mesencephalon to subcortical structures, such as the nucleus accumbens, thalamus, and the amygdala. A third pathway, the mesocortical pathway, is related to the affective and motivational dimensions of pain perception and allows the ventral tegmental area to communicate with the prefrontal cortex and the anterior cingulate cortex [30]. Lesions of the ventral tegmental area can increase sensitivity to pain, while electrical stimulation of the same area can have an analgesic effect [31]. A study using positron emission tomography found increased activation in the insula, prefrontal cortex, and anterior cingulate cortex during the “off” period [32]. All of these are areas of the limbic system associated with the affective-motivational dimension of pain [33]. Because the dopaminergic pathways and pain processing network overlap, hypofunction of the striatal dopaminergic system could result in amplification of sensory stimuli, leading to spontaneous pain sensation when dopamine levels are low [29].

Although the involvement of dopamine in central modulation of pain is well established, its role in the transmission of nociceptive stimuli from the periphery to the brain remains debatable [24]. Various studies have investigated the relationship between the threshold for pain triggered by thermal or electrical stimuli and treatment with levodopa or DBS [34–37]. Generally, the pain threshold is lower when levodopa levels are lower and tends to return to normal after administration of levodopa or after DBS even without an associated motor improvement. In other words, pain in these patients cannot be explained merely as a consequence of motor symptoms, such as rigidity, tremor, and dystonia [24].

Conte et al. [67] suggest two mechanisms in addition to striatal hypofunction to explain the different findings of their studies: degeneration of noradrenergic and serotoninergic neurons in the locus coeruleus and raphe nuclei, respectively, which can be even more pronounced than in the substantia nigra [38] and a loss of nerve fibers and Meissner corpuscles in the epidermis [71]. Together with the locus coeruleus, the gigantocellular nucleus, and the nuclei of the bulbar raphe, the periaqueductal gray matter and the parabrachial nuclei play an important role in the modulation of spinal nociceptive transmission, for example, on inhibition of nociceptive stimuli coming from the dorsal horn neuron. A disturbance in this pain-inhibiting region could cause an increase in the sensation of pain [72]. Degeneration of peripheral nociceptors also occurs in other chronic pain syndromes, such as fibromyalgia [73, 74].

Its different clinical characteristics, variable relationship with motor symptoms, and variable response to dopaminergic drugs suggest that pain in PD has a complex mechanism that involves the peripheral nervous system, the generation and amplification of pain by motor symptoms, and dopaminergic neuronal degeneration in areas responsible for central mechanisms.

## 4. The Relation between Levodopa Treatment and Pain in Parkinson's Disease

Levodopa is the most widely used and effective medication in the treatment of motor signs of PD. However, when used over a number of years, virtually all typical patients will present with complications related to pharmacokinetic and pharmacodynamic characteristics. The best-known of these include motor fluctuations such as WO, in which the therapeutic effects of levodopa do not last as long as they previously did, and dyskinesias [75]. Although only recently recognized, nonmotor fluctuations occur in a similar fashion in nearly all patients and can be main determinants of disability and worse quality of life [76]. NMS can be divided into three categories: neuropsychiatric, autonomic, and sensory. While most of these manifest during “off” periods and WO, some, such as agitation, psychosis, diaphoresis, and pain, can manifest when levodopa levels are at a peak. In this situation, pain appears to be more related to involuntary movements (dyskinesias or dystonia) than to dopamine levels per se. Fluctuation of sensory symptoms can be due to failure of primary somatosensory mechanisms [77].

Pain can be one of the main complaints reported during WO and occurs in around 50% of patients [78–80]. Chronic pain (i.e., pain lasting more than three months) is reported by more than 70% of patients with WO, suggesting that WO is a risk factor for development of chronic pain (unpublished personal data). Abdominal pain that does not respond to the use of analgesics and can only be alleviated with a new dose of levodopa can be considered a sign of WO [81, 82]. Pain that occurs only during WO can be considered of central origin, while if it is exacerbated during this period, it can be central, musculoskeletal, dystonic, or neuropathic/radicular [83].

The clinical characteristics of pain during WO and “off” periods are not well established. To our knowledge, only two studies in the literature analyzed pain during “off” periods and were only partial analyses [18, 50]. A recent study (unpublished personal data) using the McGill Pain Questionnaire found that patients with pain during WO had a higher score for the affective-motivational dimension. In the DoPaMiP study, the group with pain related to PD also had a higher score in this dimension than the pain unrelated to PD group [9]. This finding may be related to the hypothesis of dopaminergic dysfunction in the mesocorticolimbic pathway, which is associated with the affective/motivational dimension of pain [84]. In other words, degeneration of dopaminergic neurons in the ventral tegmental area may reduce pain thresholds and simultaneously activate structures in the limbic system associated with the unpleasant perception of pain.

## 5. Final Considerations

In recent years, NMS of PD have received increasing attention from physicians and researchers. Pain is present in the preclinical and initial stages of the disease and causes major impact on quality of life. Its clinical characteristics vary and can be associated with “off” periods or WO of levodopa. Although pain in PD is widespread and disabling, its clinical characteristics and pathophysiology and even the best way of classifying and treating it have yet to be defined. Further studies are required to clarify these points and elucidate the role of dopaminergic and nondopaminergic pathways in nociception, central modulation of pain, and the affective/motivational dimensions of pain perception.

## Figures and Tables

**Table 1:** Classification of pain related to Parkinson's disease.

Category	Characteristics
Musculoskeletal	Muscle and/or joint pain, inflammation, bone deformity, reduced joint mobility, and abnormal posture. Associated with muscle rigidity and can improve with levodopa therapy.

Dystonic	Associated with abnormal postures and can improve with levodopa therapy.

Neuropathic/ radicular	Peripheral neuropathic pain: restricted to the territory of the affected nerve or nerve root.

Central or primary	Neuropathic pain that is not restricted to the affected nerve or nerve root. Varies with the medication cycle as a nonmotor fluctuation. Pain may have an autonomic character, with visceral sensations. Not associated with rigidity, dystonia, or musculoskeletal or structural lesions.

Akathisia	Subjective sensation of restlessness and an inability to remain still. Can vary with medication and improve with levodopa therapy.
